# Clinical outcomes of intrathecal anti-PD-1 therapy with or without whole-brain radiotherapy in melanoma leptomeningeal metastasis: a multicenter retrospective study

**DOI:** 10.3389/fimmu.2026.1830452

**Published:** 2026-07-27

**Authors:** Junjie Zhen, Rongcheng Zhang, Dandan Li, Ya Ding, Xizhi Wen, Yanying Yang, Hui Wang, Mingyao Lai, Xiaoshi Zhang, Linbo Cai, Jingjing Li

**Affiliations:** 1 Department of Oncology, Guangdong Sanjiu Brain Hospital, Guangzhou, China; 2 State Key Laboratory of Oncology in South China, Collaborative Innovation Center for Cancer Medicine, Sun Yat-sen University Cancer Center, Guangzhou, China; 3 Department of Biological Therapy Center, Sun Yat-sen University Cancer Center, Guangzhou, China

**Keywords:** anti-PD-1 antibody, intrathecal therapy, leptomeningeal metastases, melanoma, whole-brain radiotherapy

## Abstract

**Background:**

Leptomeningeal metastases (LM) from melanoma are rare and associated with dismal outcomes. Intrathecal PD-1 antibody therapy has shown encouraging activity in LM. Radiotherapy may enhance tumor immunogenicity and potentially augment immune checkpoint blockade within the central nervous system. The efficacy and safety of combining intrathecal PD-1 antibodies with whole-brain radiotherapy (WBRT) in melanoma LM remain unclear.

**Methods:**

We retrospectively reviewed melanoma patients with LM who received intrathecal PD-1 antibody therapy between June 2022 and December 2024. Patients were categorized according to whether WBRT was administered within 30 days of intrathecal PD-1 antibody therapy: intrathecal monotherapy and combination therapy. Overall survival (OS) and intracranial progression-free survival (iPFS) were descriptively evaluated by treatment exposure, with between-group analyses considered exploratory and hypothesis-generating. Adverse events were graded using CTCAE v5.0.

**Results:**

20 patients were included, of whom 7 received intrathecal PD-1 antibody monotherapy and 13 received intrathecal PD-1 antibody therapy combined with WBRT. The observed median OS was 20.1 weeks (95% CI, 13.3-not reached) in the intrathecal monotherapy group and 45.3 weeks (95% CI, 28.7-not reached) in the combination group. The observed median iPFS was 10.0 weeks (95% CI, 6.1-not reached) and 23.0 weeks (95% CI, 18.4-not reached), respectively. Treatment-related adverse events were manageable, and no new safety signals were identified.

**Conclusion:**

Intrathecal PD-1 antibody therapy combined with WBRT showed numerically longer OS and iPFS than intrathecal therapy alone in this small retrospective descriptive series of melanoma leptomeningeal metastasis. These hypothesis-generating findings warrant prospective investigation but do not establish comparative efficacy.

## 1 Introduction

Leptomeningeal metastasis (LM) refers to the dissemination of malignant cells within the leptomeninges and the subarachnoid space (1). Approximately 10% of patients with solid tumors develop LM during the course of their disease, typically in the setting of progressive systemic involvement; in melanoma, LM accounts for roughly 5% of cases (1, 2). In addition, primary central nervous system (CNS) melanoma, a rare subtype, can also involve the leptomeninges (3). Although the clinical incidence of LM is relatively low, the prognosis remains dismal, with a median overall survival of only 6 to 10 weeks in untreated patients (4, 5).

In recent years, immune checkpoint inhibitors (ICIs), particularly programmed death-1 (PD-1) inhibitors, have shown significant efficacy in the systemic treatment of melanoma (6, 7). However, LM involves tumor cell infiltration into the subarachnoid space, creating a complex microenvironment characterized by both immune suppression and impaired drug delivery (2, 7, 8). These factors collectively limit the therapeutic effectiveness of systemically administered ICIs in melanoma patients with LM (2, 6). To address this challenge, intrathecal therapy (IT) has emerged as a promising therapeutic strategy. By bypassing the blood–brain barrier, this approach allows for the direct delivery of therapeutically relevant drug concentrations into the cerebrospinal fluid, thereby enhancing exposure at LM sites (9, 10). Preliminary data from phase I clinical trials and small retrospective studies have demonstrated the feasibility, safety, and potential clinical benefit of intrathecal PD-1 antibody administration in melanoma patients with LM (9–11). Nonetheless, the efficacy of this approach remains to be fully elucidated, particularly in the context of combination strategies with other therapeutic modalities.

Radiotherapy remains a commonly utilized modality in the management of patients with LM, offering the advantages of penetrating the blood–brain barrier—a critical limitation for certain systemic therapies (1, 12). Multiple radiotherapeutic strategies have been employed in this setting, including whole-brain radiotherapy (WBRT), stereotactic radiosurgery (SRS), craniospinal irradiation (CSI), among others (12). WBRT can alleviate neurological symptoms associated with LM and contribute to intracranial disease control (13, 14). In select melanoma patients with LM, WBRT combined with systemic therapy has been associated with improved survival outcomes (15, 16). However, its use as a monotherapy has not been definitively shown to confer a survival benefit in patients with LM (17, 18). Moreover, there is a lack of systematic evidence evaluating the efficacy of combination approaches such as WBRT with intrathecal immunotherapy in the treatment of melanoma with LM.

Therefore, we conducted a retrospective real-world study to describe clinical outcomes and treatment-related adverse events among patients with melanoma LM who received intrathecal anti-PD-1 therapy with or without WBRT. We further explored outcome patterns according to WBRT exposure to generate hypotheses for future prospective evaluation of this combination strategy.

## 2 Methods

### 2.1 Study design and patients

This retrospective study included melanoma patients with LM who received intrathecal anti-PD-1 therapy between June 2022 and December 2024. Patients were managed through multidisciplinary care at Sun Yat-sen University Cancer Center, while intrathecal interventions, including Ommaya reservoir placement and intrathecal anti-PD-1 administration, as well as WBRT when clinically indicated, were performed at Guangdong Sanjiu Brain Hospital. Patient-specific characteristics, diagnostic information, and treatment details were collected. The diagnosis of LM was primarily based on positive cerebrospinal fluid (CSF) cytology. For patients with negative CSF cytology, LM was diagnosed based on characteristic radiographic findings, such as leptomeningeal enhancement on magnetic resonance imaging, or histopathologic confirmation obtained from leptomeningeal biopsy.

### 2.2 Treatment and endpoints

Intrathecal administration was performed via a pre-implanted Ommaya reservoir or lumbar puncture, with treatment schedules consisting of nivolumab every 2 weeks or pembrolizumab every 3 weeks. For Ommaya reservoir-based delivery, the scalp was disinfected and a scalp needle was inserted into the reservoir. CSF was withdrawn slowly at a rate of approximately 1 mL/min, with a total volume of 5–10 mL. A 20 mg dose of PD-1 antibody was then diluted with normal saline to a final volume of 5 mL and slowly injected over approximately 10 minutes. Following needle removal, manual pressure was applied, and the site was disinfected and covered with a sterile dressing.

In this study, patients in the combination group received WBRT within 30 days before or after the initiation of intrathecal PD-1 antibody therapy. WBRT was delivered using intensity-modulated radiotherapy (IMRT) in all patients. The clinical target volume (CTV) encompassed the entire brain parenchyma and meningeal surfaces, including the skull base and dural reflections, with appropriate sparing of the scalp. The inferior border of the radiation field extended to the level of the second cervical vertebra to ensure full coverage of both the dura and leptomeninges. WBRT was administered in fractions of 30 Gy in 10 sessions (3 Gy per session) or 37.5 Gy in 15 sessions (2.5 Gy per session), once daily, five days per week, over a period of 2–3 consecutive weeks. SRS was administered during intrathecal treatment in selected patients with focal intracranial lesions according to individual clinical indications. In this cohort, SRS was performed in three patients, all in the intrathecal monotherapy group, for focal brain metastases or focal meningeal lesions; it was not delivered as a boost to WBRT and was not used to define treatment groups. For patients with severe intracranial hypertension and poor tolerance to radiotherapy or intrathecal therapy, ventriculoperitoneal (VP) shunt placement was considered to relieve intracranial pressure symptoms, as determined by the treating neurosurgical team. During treatment, symptoms such as increased intracranial pressure or neurological deterioration were managed primarily with osmotic agents (e.g., mannitol). Corticosteroids were not routinely administered intrathecally as part of the initial intrathecal anti-PD-1 regimen. Short-term systemic corticosteroids were permitted when clinically indicated for supportive management of pre-existing cerebral edema, increased intracranial pressure, or peri-radiotherapy neurological risk.

The primary endpoint of this study was overall survival (OS), defined as the time from the initiation of LM-directed treatment—including intrathecal PD-1 antibody administration or WBRT—to the date of death or last follow-up. The secondary endpoint was intracranial progression-free survival (iPFS), defined as the time from the initiation of LM treatment to the first radiographic evidence of intracranial progression or death, whichever occurred first. LM status was assessed using an integrated framework informed by RANO-LM criteria, incorporating neurological examination, CNS imaging, and CSF cytology when available (19). Given the recognized limitations of CSF cytology in sensitivity, reproducibility, and quantitative monitoring, clinical/radiographic improvement was interpreted descriptively, primarily based on neurological assessment and CNS imaging (20). Adverse events (AEs) were initially assessed by the treating physician at the time of occurrence, followed by independent review, quality control, and attribution by a medical oncologist. The severity of treatment-related AEs was graded according to the National Cancer Institute Common Terminology Criteria for Adverse Events (CTCAE), version 5.0.

### 2.3 Statistical analysis

Categorical variables were summarized as frequencies and percentages, and continuous variables were summarized as medians with interquartile ranges (IQRs). Baseline clinical and treatment characteristics were descriptively summarized according to treatment exposure. Given the small sample size and non-randomized treatment assignment, formal statistical comparisons of baseline characteristics were not used to assess group comparability. The Kaplan-Meier method was used to estimate the distribution of OS and iPFS from the initiation of the study treatment. The median OS and iPFS with 95% confidence intervals (CIs) were also reported according to treatment exposure.

Univariable Cox proportional hazards regression was performed as an exploratory analysis to estimate associations between selected clinical characteristics, including treatment exposure, and OS. Given the limited sample size and the risk of model overfitting, multivariable Cox regression analyses were not used to draw conclusions regarding the comparative efficacy of combination therapy versus intrathecal therapy alone. In cases where the proportional hazards assumption was violated, restricted mean survival time (RMST)—a non-parametric measure defined as the area under the survival curve up to a prespecified time point—was employed. Given the potential violation of the proportional hazards assumption, a secondary analysis using RMST at the 1-year time point was prespecified for OS as an exploratory analysis. RMST was not applied to iPFS because iPFS was evaluable only in patients with available post-treatment imaging follow-up. All statistical tests used in exploratory analyses were two-sided, and corresponding P values were interpreted descriptively rather than as evidence of definitive treatment differences. Statistical analyses and visualizations were performed using R version 4.2.2 and GraphPad Prism version 10.3.1.

## 3 Results

### 3.1 Patients and disease characteristics

This retrospective observational study included 20 patients with leptomeningeal metastases from malignant melanoma who received intrathecal PD-1 antibody therapy between June 2022 and December 2024. For descriptive analyses according to treatment exposure, patients were categorized into an intrathecal monotherapy group (n = 7, 35.0%) and a combination therapy group comprising patients who received whole-brain radiotherapy (WBRT) within 30 days before or after intrathecal treatment (n = 13, 65.0%). Ventriculoperitoneal shunting for clinically significant intracranial hypertension was performed in 4 patients, all in the combination therapy group; timing relative to intrathecal anti-PD-1 therapy is summarized in **Supplementary Table 4**.

Clinical and treatment characteristics according to treatment exposure are summarized in **Table 1**. Cutaneous melanoma was the most common primary subtype, accounting for 5 of 7 patients (71.4%) in the intrathecal monotherapy group and 5 of 13 patients (38.5%) in the combination therapy group. Primary central nervous system (CNS) melanoma was present in 1 patient (14.3%) in the intrathecal monotherapy group and 5 patients (38.5%) in the combination therapy group. BRAF V600E/K mutations were present in 4 patients (57.1%) of the intrathecal monotherapy group and 4 patients (30.8%) of the combination therapy group. Brain parenchymal metastases were observed in 5 patients (71.4%) receiving intrathecal monotherapy and 10 patients (76.9%) receiving combination therapy, while positive CSF cytology was documented in 6 patients (85.7%) and 9 patients (69.2%), respectively. Most patients had received prior systemic therapy, with a median of 3.0 and 1.0 lines of treatment in the intrathecal monotherapy and combination therapy groups, respectively. Intrathecal PD-1 antibody was administered primarily via an Ommaya reservoir in both groups, with nivolumab being the most frequently used agent. Prior immune checkpoint inhibitor exposure before intrathecal therapy is summarized in **Table 1**. In the intrathecal monotherapy group, 6 patients (85.7%) had prior anti-PD-1 exposure, all as anti-PD-1 monotherapy, and 1 patient (14.3%) was anti-PD-1 naïve. In the combination therapy group, 8 patients (61.5%) had prior anti-PD-1 exposure, including 7 (53.8%) who had received anti-PD-1 monotherapy and 1 (7.7%) who had received combined anti-PD-1 plus anti-CTLA-4 blockade, while 5 patients (38.5%) were anti-PD-1 naïve. No patient in either group had received anti-CTLA-4 monotherapy alone before intrathecal therapy.

**Table 1:** Clinical and treatment characteristics according to treatment exposure.

Characterisic	Group	
	IT N = 7	IT+WBRT N = 13*
Age		
Median (Q1, Q3)	30 (29, 51)	36 (27, 56)
Sex, n (%)		
Female	5 (71.4)	6 (46.2)
Male	2 (28.6)	7 (53.8)
KPS		
Median (Q1, Q3)	80 (70, 80)	70 (60, 80)
Primary melanoma subtype, n (%)		
Cutaneous	5 (71.4)	5 (38.5)
Acral	1 (14.3)	1 (7.7)
Mucosal	0 (0)	2 (15.4)
CNS	1 (14.3)	5 (38.5)
BRAF V600E/K mutation, n (%)		
No	3 (42.9)	9 (69.2)
Yes	4 (57.1)	4 (30.8)
Brain parenchymal metastases, n (%)		
No	2 (28.6)	3 (23.1)
Yes	5 (71.4)	10 (76.9)
CSF cytology, n (%)		
Negative	1 (14.3)	4 (30.8)
Positive	6 (85.7)	9 (69.2)
Histopathologic confirmation by leptomeningeal biopsy, n (%)		
Negative	2 (28.6)	3 (23.1)
Positive	2 (28.6)	2 (15.4)
Not performed	3 (42.9)	8 (61.5)
Lines of systemic treatments at diagnosis of LM		
Median (Q1, Q3)	3 (1, 5)	1 (1, 3)
Method of intrathecal injection, n (%)		
Ommaya reservoir	5 (71.4)	12 (92.3)
Lumbar puncture	1 (14.3)	0 (0)
Both	1 (14.3)	1 (7.7)
Intrathecal PD-1		
Nivolumab 20 mg every 2 weeks	6 (85.7)	11 (84.6)
Pembrolizumab 20 mg every 3 weeks	1 (14.3)	2 (15.4)
Prior ICI exposure before diagnosis of LM, n (%)		
Anti-PD-1 monotherapy	6 (85.7)	7 (53.8)
Anti-CTLA-4 monotherapy	0	0
Anti-PD-1 plus anti-CTLA-4 combination	0	1 (7.7)
No prior immunotherapy	1 (14.3)	5 (38.5)

*Among all 13 patients, two were diagnosed with LM based solely on characteristic MRI findings. IT, Intrathecal Therapy; WBRT, Whole Brain Radiotherapy; CNS, central nervous system; CSF, cerebrospinal fluid; LM, leptomeningeal metastasis; ICI, immune checkpoint inhibitor.

### 3.2 Clinical outcomes and treatment response

**Figure 1:**
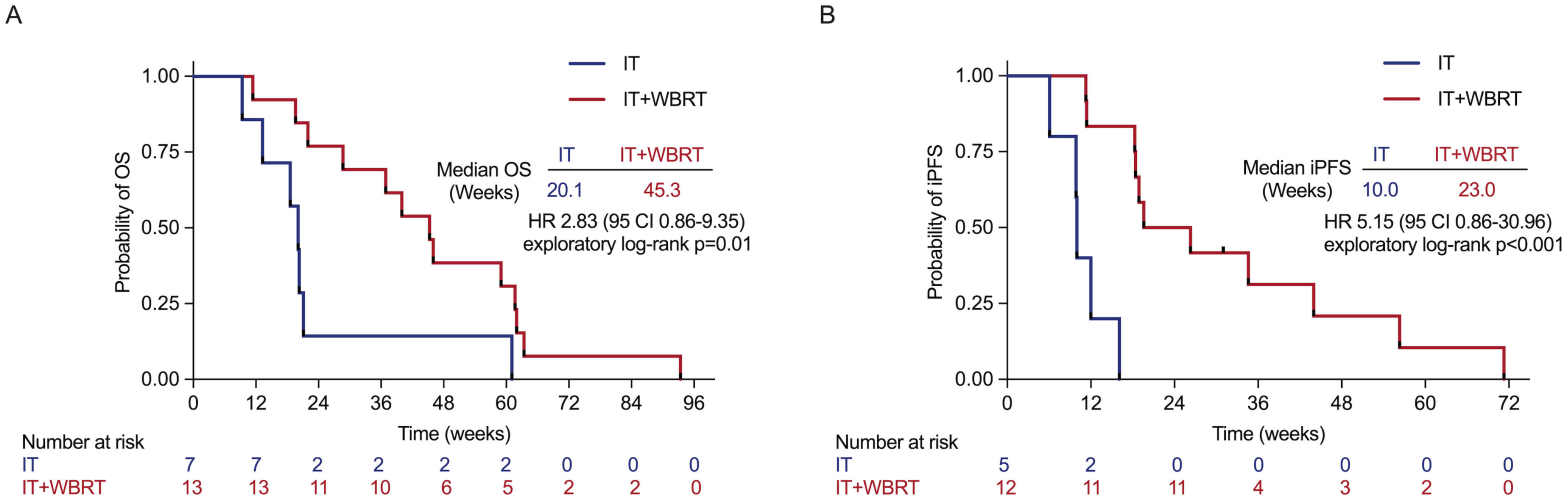
Observed survival outcomes according to treatment exposure. Kaplan–Meier curves show **(A)** OS and **(B)** iPFS among patients receiving intrathecal anti-PD-1 therapy alone or in combination with WBRT. Median OS and iPFS were numerically longer in the combination therapy group. Log-rank P values, where shown, represent exploratory between-group analyses and should not be interpreted as confirmatory evidence of comparative efficacy. Abbreviations: WBRT, whole-brain radiotherapy; OS, overall survival; iPFS, intracranial progression-free survival.

By the data cutoff of August 15, 2025, all patients had experienced the OS event, and the median follow-up duration was 32.8 weeks (IQR, 19.9 - 60.1). Median OS was 45.3 weeks (95% CI, 28.7 - not reached) in the combination therapy group and 20.1 weeks (95% CI, 13.3 - not reached) in the intrathecal monotherapy group (exploratory P = 0.01) (**Figure 1A**). In an exploratory RMST analysis up to 1 year, the estimated mean survival time was 39.2 weeks (95% CI, 31.7 - 46.7) in the combination therapy group and 22.1 weeks (95% CI, 12.6 - 31.6) in the intrathecal monotherapy group, corresponding to an observed difference of 17.1 weeks. Patients in the combination therapy group received a higher median number of intrathecal PD-1 antibody treatment cycles than those in the monotherapy group (6.0 vs. 4.0 cycles). Exploratory univariable associations between selected clinical characteristics and OS are shown in **Supplementary Figure 1** For iPFS, two patients in the intrathecal monotherapy group and one patient in the combination group were not evaluable due to lack of post-treatment imaging follow-up. Among the 17 evaluable patients, median iPFS was 23.0 weeks (95% CI, 18.4 - not reached) in the combination therapy group and 10.0 weeks (95% CI, 6.1 - not reached) in the intrathecal monotherapy group (exploratory P < 0.001) (**Figure 1B**).

**Figure 2:**
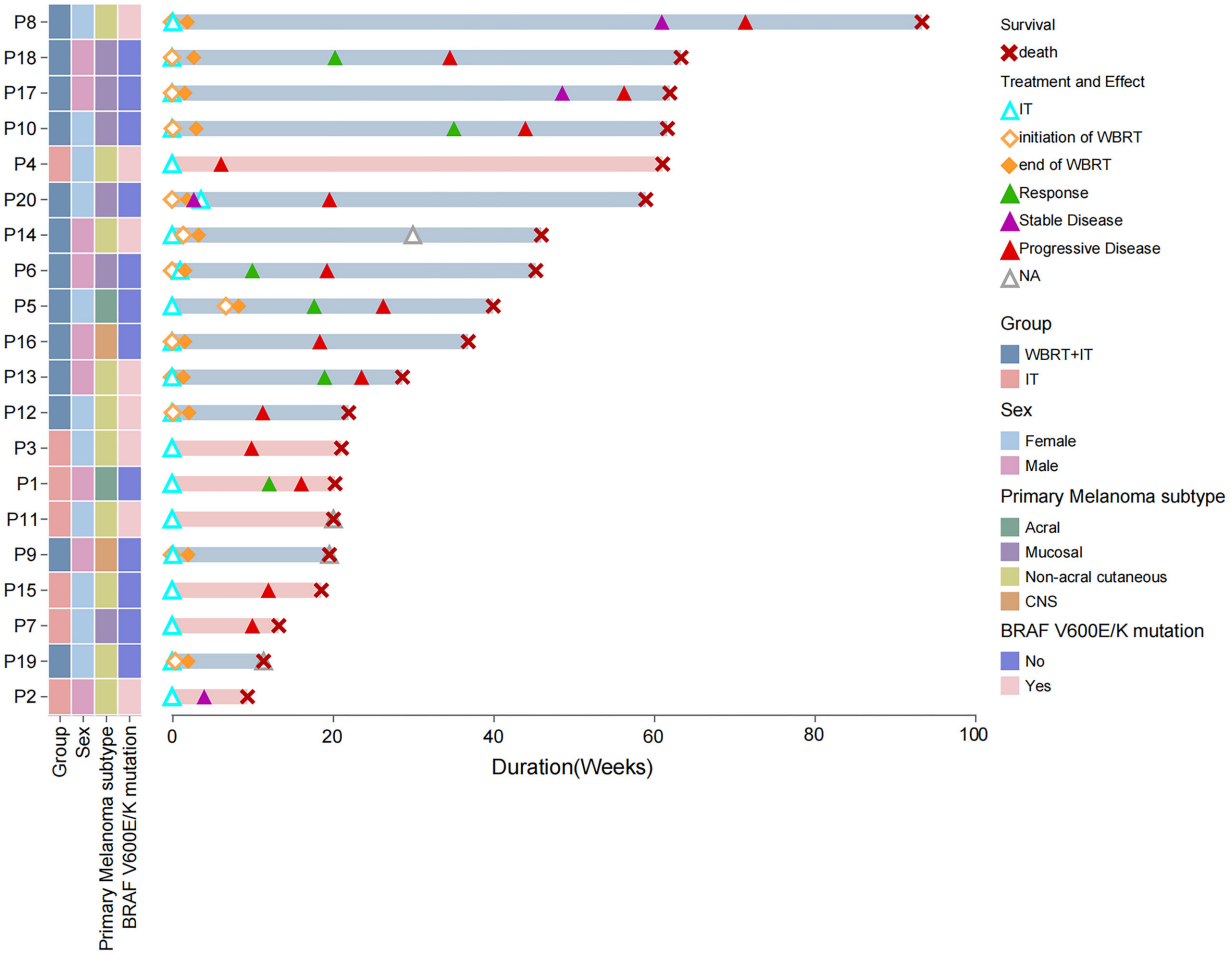
Swimmer plot summarizing individual treatment courses, responses, and outcomes. Each horizontal bar represents a single patient, depicting the duration from the initiation of LM-directed treatment—including intrathecal PD-1 antibody administration or WBRT—to the date of death or last follow-up (weeks). Red crosses mark death. Orange and blue segments denote timing of WBRT and intrathecal therapy, respectively. Symbols on each bar indicate key clinical events, including initiation/completion of WBRT, best LM status and subsequent documented progression. Patients are grouped by treatment cohort (WBRT + IT vs IT alone). Additional annotations (left) indicate sex, melanoma subtype, and BRAF V600E/K mutation status. WBRT, whole-brain radiotherapy; IT, intrathecal therapy.

Best LM status was assessed descriptively based primarily on neurological examination and CNS imaging, with CSF cytology considered when available. Progression events were adjudicated using RANO-LM-defined worsening criteria, and patient-level supporting findings are summarized in **Supplementary Table 2**. In the intrathecal monotherapy group, one patient demonstrated a radiographic response, one had stable leptomeningeal disease, four experienced disease progression during treatment, and one patient was not evaluable. In the combination therapy group, five patients showed a radiographic response, three had stable disease, two experienced progression, and three patients were not evaluable. Selected clinical characteristics, treatment courses, and response assessments are summarized in **Figure 2**. In most patients in the combination therapy group, WBRT was initiated concurrently with IT (within 24 hours). Two patients received WBRT prior to intrathecal injection, and two received IT before WBRT; in both cases, the interval between modalities was ≤ 30 days. In addition to LM-directed therapy, patients also received concurrent systemic treatment, with detailed regimens listed in **Supplementary Table 1**. Accordingly, the observed outcomes reflect multimodal real-world treatment exposure rather than the isolated effect of WBRT added to intrathecal PD-1 antibody therapy.

When stratified by prior ICI exposure before intrathecal therapy, documented progression during or after intrathecal treatment was observed in 1 of 6 patients (16.7%) without prior immunotherapy and in 5 of 14 patients (35.7%) with prior ICI exposure. We further performed an exploratory survival analysis restricted to patients with prior ICI exposure. In this subgroup, the combination therapy group showed numerically longer median OS (38.5 vs. 20.2 weeks) and, among patients evaluable for iPFS, numerically longer median iPFS (26.3 vs. 11.0 weeks) than the intrathecal monotherapy group, although this analysis was limited by very small numbers (**Supplementary Figure 2**).

### 3.3 Safety

Treatment-related AEs were observed in 5 patients (71.4%) in the intrathecal monotherapy group and 4 patients (30.8%) in the combination therapy group. All AEs were grade 1–2, and no grade ≥3 events were reported. The detailed profiles and grades of adverse events in both groups are summarized in **Table 2**. In the intrathecal monotherapy group, AEs included vomiting (n = 1, 14.3%), headache (n = 3, 42.9%), and lower back pain (n = 1, 14.3%). In the combination therapy group, AEs were primarily vomiting (n = 2, 15.4%) and bone marrow suppression (n = 2, 15.4%). All adverse events resolved spontaneously during hospitalization or were managed effectively with symptomatic treatment. During treatment, baseline symptoms such as increased intracranial pressure or neurological deterioration were managed primarily with osmotic agents (e.g., mannitol). To prevent severe radiation-induced edema exacerbation—particularly in patients with high baseline intracranial pressure or peritumoral edema associated with parenchymal brain metastases—short courses of low-dose prophylactic corticosteroids (e.g., dexamethasone) were administered peri-radiotherapy. Intrathecal dexamethasone was used in only two non-routine situations: together with intrathecal methotrexate as salvage treatment after progression on intrathecal anti-PD-1 therapy in one patient, and together with intrathecal vancomycin for treatment-emergent intracranial infection in another. However, long-term routine use of immunosuppressive corticosteroids was strictly avoided whenever possible to minimize interference with PD-1 blockade efficacy. Detailed corticosteroid regimens, including type, dose, and indication, are summarized in the **Supplementary Table 3**.

**Table 2:** Recorded treatment-related adverse events according to treatment exposure.

Adverse events N (%)	Group	
	IT N=7	WBRT+IT N=13
Vomiting	1 (14.3)	2 (15.4)
Grade 1	0	1 (7.7)
Grade 2	1 (14.3)	1 (7.7)
Myelosuppression	0	2 (15.4)
Grade 1	0	1 (7.7)
Grade 2	0	1 (7.7)
Headache	3 (42.9)	0
Grade 1	1 (14.3)	0
Grade 2	2 (28.6)	0
Low back pain	1 (14.3)	0
Grade 1	1 (14.3)	0
Grade 2	0	0

IT, Intrathecal Therapy; WBRT, Whole Brain Radiotherapy.

## 4 Case illustration

**Figure 3:**
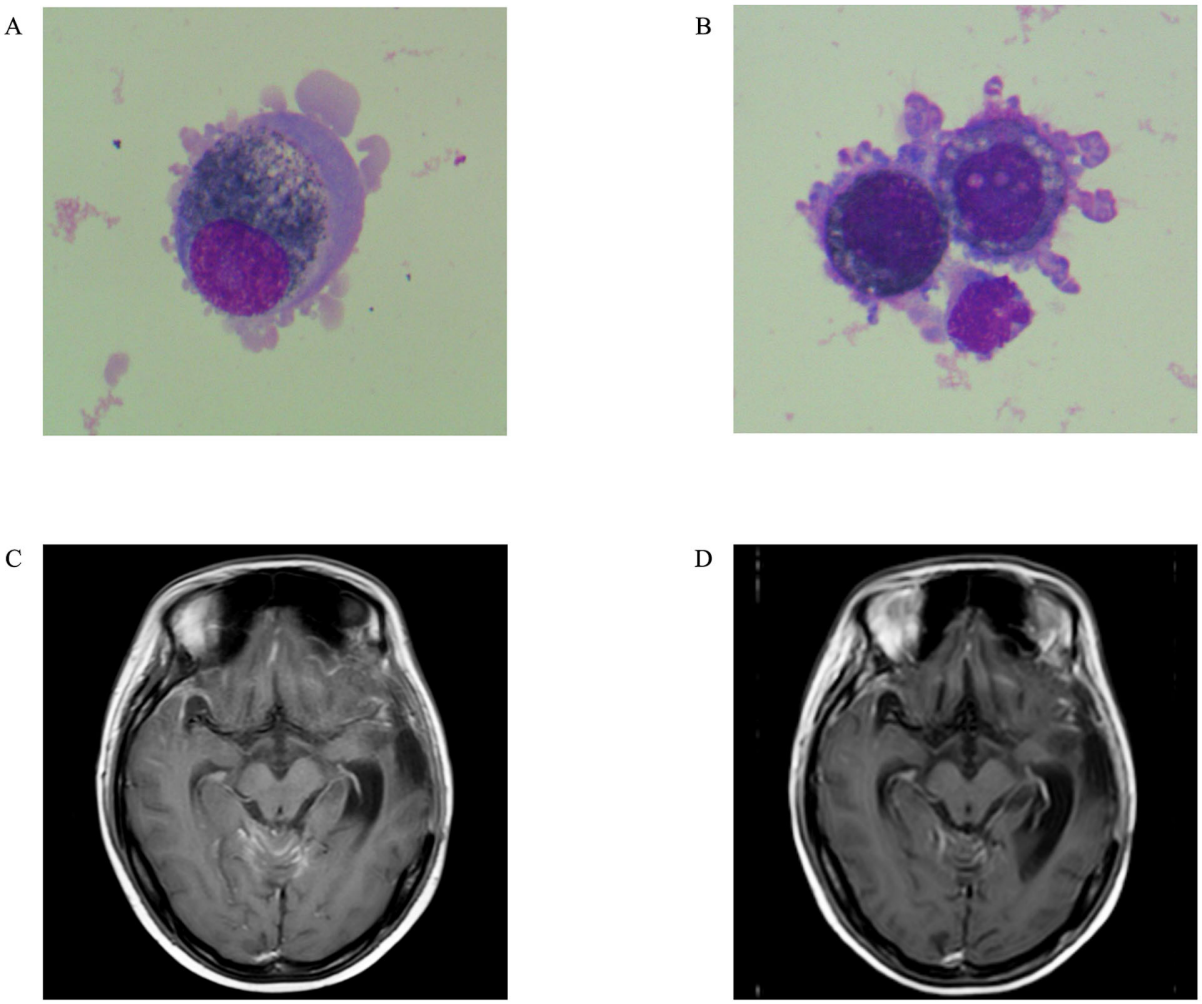
Cytopathologic and radiographic findings in a representative patient with melanoma leptomeningeal metastasis. Cerebrospinal fluid cytology demonstrating malignant melanoma cells before intrathecal anti-PD-1 treatment **(A, B)**. MRI findings of patient 10 before intrathecal anti-PD-1 treatment **(C)** and reduction of leptomeningeal lesions **(D)**.

Patient 10, a 27-year-old female, presented with recurrent headaches and was found to have a left temporal lobe mass on brain MRI. Surgical resection was performed, and pathological analysis confirmed malignant melanoma with an NRAS Q61 mutation and PD-L1 expression of 3%. PET/CT revealed no extracranial lesions. The patient received temozolomide chemotherapy combined with pembrolizumab following surgery, as well as intensity-modulated radiation therapy (IMRT, 45 Gy/10 fractions) targeting the surgical cavity. Following disease progression, treatment was switched to nab-paclitaxel, carboplatin, and bevacizumab. After six cycles, she developed worsening headache, nausea, and vomiting. CSF cytology revealed malignant melanoma cells (**Figures 3A, B**), and MRI showed diffuse leptomeningeal and spinal meningeal enhancement on T1-weighted images (**Figure 3C**), confirming leptomeningeal metastasis. After multidisciplinary assessment, an Ommaya reservoir was implanted. She received WBRT (37.5 Gy/15 fractions) along with intrathecal administration of nivolumab (20 mg every 2 weeks, 2 cycles). Subsequently, she continued intrathecal PD-1 therapy in combination with systemic treatment including pembrolizumab (200 mg), ipilimumab (50 mg), and dacarbazine (DTIC, 0.6 g on days 1–2). After 10 cycles of IT, brain MRI demonstrated marked reduction in leptomeningeal enhancement (**Figure 3D**), and neurological symptoms improved, with a clinical response categorized as improved based on clinical and radiographic assessment. However, after 12 cycles, the patient developed worsening neurological symptoms, including headache and seizures, and follow-up MRI indicated disease progression with increased leptomeningeal enhancement. The patient eventually succumbed to intracranial tumor progression after completing 14 cycles of intrathecal PD-1 therapy. Her iPFS was 24.0 weeks, and OS from the diagnosis of leptomeningeal disease was 46.1 weeks.

## 5 Discussion

To our knowledge, this study represents one of the earlier real-world series describing clinical outcomes among melanoma patients with LM who received intrathecal anti-PD-1 therapy with or without WBRT. Patients in the combination group had numerically longer observed OS and iPFS than those in the intrathecal monotherapy group, and the direction of this observation was consistent in exploratory Cox and RMST analyses. No grade ≥3 treatment-related adverse events or toxicity-related treatment discontinuations were observed. However, since the study was not randomized to WBRT and the sample size was small, with heterogeneity in baseline and concurrent treatments, these findings should be interpreted as descriptive.

In recent years, systemic ICIs have been shown to confer significant survival benefits in patients with metastatic brain melanoma (MBM) (21). However, nearly all prospective clinical trials have excluded patients with LM (7), leaving a major evidence gap and creating substantial challenges in clinical management. To date, only a limited number of case reports and retrospective studies have evaluated the efficacy of systemic ICI therapy in patients with melanoma-associated LM, but none have demonstrated a clear clinical benefit (11, 15, 22, 23). Intrathecal administration of ICIs is increasingly recognized as a viable approach to overcome the poor CNS and CSF penetration of systemically delivered monoclonal antibodies (9, 24). According to Glitza Oliva et al., a preliminary phase 1 study demonstrated that intrathecal nivolumab, administered concurrently with intravenous nivolumab, resulted in a median overall survival of 4.9 months in patients with leptomeningeal metastases, including those with melanoma, most of whom were previously resistant to PD-1 inhibitors (10). In our study, the median OS for patients receiving intrathecal PD-1 antibody therapy without WBRT was 20.1 weeks, which is comparable to the outcomes reported by Glitza Oliva et al. This cross-study comparison is contextual only, given differences in patient selection, prior treatments, concomitant therapies, and study design. Furthermore, the clinical heterogeneity of the present cohort, including differences in melanoma subtype and prior systemic treatment exposure, precludes conclusions regarding the relative activity of intrathecal anti-PD-1 therapy across specific melanoma subtypes.

Radiotherapy has long been a cornerstone in the management of patients with LM. In Europe, focal involved-field radiotherapy (IFRT) is often preferred for treating discrete intracranial and spinal levels. However, given that LM frequently disseminate throughout the neuroaxis, focal irradiation alone is insufficient to address the full extent of disease, resulting in limited survival benefit (20). While several studies have reported an association between WBRT and prolonged survival (25, 26), other data have failed to demonstrate a statistically significant impact of WBRT on OS (13, 27). In current clinical practice, WBRT is used primarily for palliation—relieving symptoms and controlling symptomatic lesions (1). In recent years, concerns regarding neurocognitive decline have led to a decline in the routine use of WBRT for LM. Nevertheless, WBRT remains valuable in selected scenarios because it can comprehensively treat multifocal disease and subclinical leptomeningeal involvement (12, 20, 28).

Emerging evidence also supports potential synergy between radiotherapy and immunotherapy. Radiotherapy can induce immunogenic cell death, promoting the release of tumor-associated antigens and enhancing dendritic cell activation and T-cell priming. These effects collectively increase the immunogenicity of the tumor microenvironment (6, 12, 24). Preclinical studies and early-phase clinical trials have demonstrated that radiation-induced tumor antigen release can enhance the targeting efficacy of PD-1 antibodies within the leptomeningeal compartment (29). In addition, WBRT may enhance antitumor immune sensitivity by upregulating major histocompatibility complex class I (MHC-I) expression on tumor cells within the central nervous system, thereby increasing tumor antigen presentation and augmenting immune responsiveness following PD-1/PD-L1 pathway blockade (30, 31). Concurrently, immunotherapy relieves immunosuppression, thereby sustaining and enhancing radiation-induced inflammation and T-cell infiltration, which in turn improves the efficacy of radiotherapy and helps suppress local recurrence (32, 33). Recent retrospective evidence has indicated that combining WBRT with systemic PD-1 blockade may enhance therapeutic efficacy in melanoma patients with LM (34).

On this basis, combining WBRT with intrathecal anti-PD-1 therapy is a clinically relevant strategy to explore: WBRT may provide broader local treatment of intracranial leptomeningeal disease, while intrathecal anti-PD-1 therapy may directly engage the cerebrospinal fluid compartment. In the present cohort, patients receiving this combination had observed median OS and iPFS of 45.3 weeks and 23.0 weeks, respectively, compared with 20.1 weeks and 10.0 weeks among patients receiving intrathecal therapy alone. Although these numerically favorable outcomes are compatible with the proposed rationale for combined treatment, they cannot establish therapeutic synergy or an additive benefit of WBRT because treatment assignment was non-randomized, patients received heterogeneous concomitant systemic therapies, and clinically relevant differences existed between exposure groups. These observations support prospective evaluation of the combination strategy rather than definitive clinical adoption on the basis of comparative efficacy.

The safety observations in this cohort were generally manageable. All recorded treatment-related adverse events were grade 1-2, and no grade ≥3 treatment-related adverse events or treatment discontinuations due to toxicity were observed in either exposure group. In the intrathecal monotherapy group, the most frequently recorded adverse event was headache, whereas vomiting and bone marrow suppression were recorded in the combination therapy group. Corticosteroids were not routinely administered intrathecally with anti-PD-1 therapy; when required, they were predominantly administered intravenously as short-term supportive management for pre-existing cerebral edema or increased intracranial pressure during radiotherapy. Intrathecal dexamethasone was limited to non-routine clinical situations and was not part of the initial intrathecal anti-PD-1 protocol. Since supportive corticosteroids were frequently used, the absence of recorded severe acute edema-related events should be interpreted in this context. Meanwhile, formal neurocognitive assessments were not routinely performed, and thus the delayed neurocognitive toxicity could not be evaluated. These findings therefore provide descriptive tolerability information rather than evidence that the addition of WBRT carries no incremental toxicity.

This study is limited by its retrospective design, small sample size, non-randomized administration of WBRT, and heterogeneity in baseline characteristics and concomitant systemic treatments. Prior ICI exposure may also have influenced treatment response and outcome interpretation. Documented progression during or after intrathecal treatment was observed in 1 of 6 patients without prior immunotherapy and in 5 of 14 patients with prior ICI exposure. Patients without prior immunotherapy were more common in the combination therapy group than in the intrathecal monotherapy group at baseline, and the only progression event among patients without prior immunotherapy occurred in the intrathecal monotherapy group. This imbalance makes it difficult to separate the potential contribution of anti-PD-1–naïve status from that of WBRT exposure. To further explore this issue, we performed an exploratory analysis restricted to patients with prior ICI exposure. Although limited by very small numbers, this analysis still showed numerically longer OS and iPFS in the combination therapy group, with a direction consistent with the overall cohort. These findings suggest that the numerically favorable outcomes in the combination group were not solely driven by the higher proportion of anti-PD-1–naïve patients. Nevertheless, given the retrospective design, small subgroup size, and non-randomized treatment assignment, this observation should be interpreted only as hypothesis-generating and cannot establish an independent benefit of WBRT added to intrathecal anti-PD-1 therapy.

Moreover, systemic anti-PD-1 therapy was administered concurrently during LM-directed treatment in a substantial proportion of patients, particularly in the combination therapy group, limiting attribution of observed outcomes to intrathecal anti-PD-1 delivery or WBRT exposure alone. Accordingly, the observed survival differences and exploratory Cox and RMST findings cannot establish a comparative benefit of the combination strategy. Because iPFS was evaluable only in patients with available post-treatment imaging follow-up, the iPFS analysis may be subject to evaluability bias. Although all patients receiving WBRT were treated using IMRT, two dose-fractionation schedules were used, and the small sample size precluded subgroup analyses according to dose schedule. While our findings demonstrate numerically favorable survival outcomes and safety, the absence of long-term follow-up precludes conclusions regarding durable efficacy and neurocognitive outcomes. Large-scale, prospective, randomized controlled trials are warranted to validate these findings and to refine treatment strategies for this challenging population.

## 6 Conclusions

In this retrospective real-world series of melanoma patients with LM, intrathecal anti-PD-1 therapy combined with WBRT was associated with numerically favorable OS and iPFS outcomes compared with intrathecal therapy alone, with no recorded grade ≥3 treatment-related AEs. Given the small sample size, non-randomized treatment exposure, clinical heterogeneity, and concomitant therapies, these findings should be interpreted as descriptive and hypothesis-generating. Prospective studies are warranted to determine whether the addition of WBRT provides meaningful clinical benefit and acceptable long-term tolerability.

## Data Availability

The raw data supporting the conclusions of this article will be made available by the authors, without undue reservation.
