# Supplementary material for: Clinical outcomes of intrathecal anti-PD-1 therapy with or without whole-brain radiotherapy in melanoma leptomeningeal metastasis: a multicenter retrospective study
Source: Front Immunol. 2026 Jul 27;17:1830452. doi: 10.3389/fimmu.2026.1830452 (PMC13454150; doi:10.3389/fimmu.2026.1830452)
Supplement: Supplementary file 1 [file DataSheet1.pdf]

Supplementary Materials

Supplementary Table 1. Detailed information of patient disease characteristics and treatment

Patient ID	Group	Systemic treatment lines at LM diagnosis	Prior ICI exposure by the time of LM diagnosis	Concurrent systemic therapies during IT treatment (After LM diagnosis)	Response of extracranial lesions during LM treatment	Best LM response during treatment
1	IT	3	Anti-PD-1 monotherapy	Nivolumab, Nab-paclitaxel, Bevacizumab	Progression	Response
2	IT	5	Anti-PD-1 monotherapy	Dabrafenib, Trametinib	Progression	Stable Disease
3	IT	3	Anti-PD-1 monotherapy	Dabrafenib, Trametinib	Non-Progression	Progressive Disease
4	IT	1	Anti-PD-1 monotherapy	Pembrolizumab, Ipilimumab, Dabrafenib, Trametinib	Non-Progression	Progressive Disease
5	IT+WBRT	3	Anti-PD-1 monotherapy	Pembrolizumab, Bevacizumab	Progression	Response
6	IT+WBRT	0	No prior immunotherapy	Pembrolizumab, Ipilimumab, Nab-paclitaxel, Temozolomide	Non-Progression	Response
7	IT	0	No prior	Pembrolizumab,	Non-Progression	Progressive Disease

			immunotherapy	Ipilimumab, Temozolomide		
8	IT+WBRT	2	Anti-PD-1 monotherapy	Toripalimab, Dabrafenib, Trametinib	Progression	Stable Disease
9	IT+WBRT	1	Anti-PD-1 monotherapy	NA	Non-Progression	NA
10	IT+WBRT	3	Anti-PD-1 monotherapy	Pembrolizumab, Ipilimumab, Temozolomide, Dacarbazine	Non-Progression	Response
11	IT	5	Anti-PD-1 monotherapy	NA	Non-Progression	NA
12	IT+WBRT	3	Anti-PD-1 monotherapy	NA	Non-Progression	Progressive Disease
13	IT+WBRT	1	No prior immunotherapy	Dabrafenib, Trametinib	Non-Progression	Response
14	IT+WBRT	1	No prior immunotherapy	Toripalimab, Ipilimumab, Dabrafenib, Trametinib	Progression	NA
15	IT	1	Anti-PD-1 monotherapy	Temozolomide	Progression	Progressive Disease
16	IT+WBRT	1	Anti-PD-1 monotherapy	Toripalimab, Nab-paclitaxel, Bevacizumab, Temozolomide	Progression	Progressive Disease
17	IT+WBRT	1	Anti-PD-1 monotherapy	Tislelizumab, Temozolomide	Non-Progression	Stable Disease

18	IT+WBRT	1	No prior immunotherapy	Toripalimab, Temozolomide	Non-Progression	Response
19	IT+WBRT	5	Anti-PD-1 plus anti-CTLA-4 combination	Toripalimab, Temozolomide	Non-Progression	NA
20	IT+WBRT	0	No prior immunotherapy	Nivolumab, Nab-paclitaxel, Bevacizumab	Non-Progression	Stable Disease

IT, Intrathecal Therapy; WBRT, Whole Brain Radiotherapy; LM, Leptomeningeal Metastases; ICI, immune checkpoint inhibitor; NA, Not Available.

Supplementary Table 2. Specific RANO-LM progression criteria for patients with documented progression

Patient ID	Group	Neurological Exam	CSF cytology finding	CNS imaging
3	IT	Stable	Persistent Positive	Worse
4	IT	Stable	Persistent Positive	Worse
7	IT	Worse	Persistent Positive	Worse
12	IT+WBRT	Worse	Persistent Positive	Worse
15	IT	Worse	Persistent Positive	Worse
16	IT+WBRT	Stable	Persistent Negative	Worse

CSF, cerebrospinal fluid; CNS, central nervous system; IT, Intrathecal Therapy; WBRT, Whole Brain Radiotherapy.

Supplementary Table 3. Patient corticosteroid usage profile

Patient ID	Group	Corticosteroid type	Times (Day)	Dose (d^{-1})	Route of administration	Indication
1	IT	dexamethasone	3	2mg	IV	Short-term prophylaxis against cerebral edema following SRS for brain metastases.
2	IT	NA	0	NA	NA	NA
3	IT	dexamethasone	4	2mg	IV	Short-term prophylaxis against cerebral edema following SRS for brain metastases.
4	IT	dexamethasone	5	2mg	IV	To alleviate cerebral edema after SRS targeting focal meningeal lesions.
5	IT+WBRT	dexamethasone	3	2.5mg	IV	Pre-transfusion processing.
6	IT+WBRT	dexamethasone	6	2-5mg	IT	Intrathecal injection of dexamethasone and methotrexate **after progression** on intrathecal anti-PD-1.
7	IT	dexamethasone	20	1-7.5mg	IT	1. Intrathecal vancomycin combined with dexamethasone was administered to manage **intracranial infection***. 2. Dexamethasone was also used to relieve neurological symptoms such as headache.
8	IT+WBRT	NA	0	NA	NA	NA
9	IT+WBRT	dexamethasone	2	7	IV	To alleviate edema after cranial radiotherapy.
10	IT+WBRT	dexamethasone	4	12	IV	To reduce post-radiotherapy cerebral edema and relieve intracranial hypertension symptoms.
11	IT	NA	0	NA	NA	NA
12	IT+WBRT	dexamethasone	5	7	IV	To reduce post-radiotherapy cerebral edema and relieve intracranial hypertension symptoms.
13	IT+WBRT	dexamethasone	5	8	IV	To reduce post-radiotherapy cerebral edema and relieve intracranial hypertension symptoms.

14	IT+WBRT	dexamethasone	2.5	8	IV	To reduce post-radiotherapy cerebral edema and relieve intracranial hypertension symptoms.
15	IT	NA	0	NA	NA	NA
16	IT+WBRT	dexamethasone	5	3	IV	To reduce post-radiotherapy cerebral edema and relieve intracranial hypertension symptoms.
17	IT+WBRT	dexamethasone	5	8	IV	To reduce post-radiotherapy cerebral edema and relieve intracranial hypertension symptoms.
18	IT+WBRT	dexamethasone	5	10	IV	To reduce post-radiotherapy cerebral edema and relieve intracranial hypertension symptoms.
19	IT+WBRT	dexamethasone	2.5	5	IV	To reduce post-radiotherapy cerebral edema and relieve intracranial hypertension symptoms.
20	IT+WBRT	dexamethasone	5	5	IV	To reduce post-radiotherapy cerebral edema and relieve intracranial hypertension symptoms.

* Ommaya reservoir-associated CNS infection with fever and positive CSF bacterial culture. Intrathecal vancomycin via the Ommaya reservoir was administered after inadequate response to intravenous vancomycin. IT, Intrathecal Therapy; WBRT, Whole Brain Radiotherapy; IV, Intravenous infusion; NA, Not Available.

Supplementary Table 4. Ventriculoperitoneal shunting in the study cohort

Patient ID	Group	VP shunt	Indication	Timing relative to IT therapy
1	IT	No	NA	NA
2	IT	No	NA	NA
3	IT	No	NA	NA
4	IT	No	NA	NA
5	IT+WBRT	No	NA	NA
6	IT+WBRT	Yes	Intracranial hypertension	Before IT
7	IT	No	NA	NA
8	IT+WBRT	No	NA	NA
9	IT+WBRT	No	NA	NA
10	IT+WBRT	No	NA	NA
11	IT	No	NA	NA
12	IT+WBRT	Yes	Persistent intracranial hypertension	After 2 cycles of IT
13	IT+WBRT	Yes	Intracranial hypertension	Before IT
14	IT+WBRT	No	NA	NA
15	IT	No	NA	NA
16	IT+WBRT	No	NA	NA
17	IT+WBRT	No	NA	NA
18	IT+WBRT	Yes	Intracranial hypertension	Before IT
19	IT+WBRT	No	NA	NA
20	IT+WBRT	No	NA	NA

IT, Intrathecal Therapy;WBRT, Whole Brain Radiotherapy;VP, ventriculoperitoneal;NA, Not Available.

Supplementary Figure 1. Exploratory univariable Cox regression analysis of overall survival.

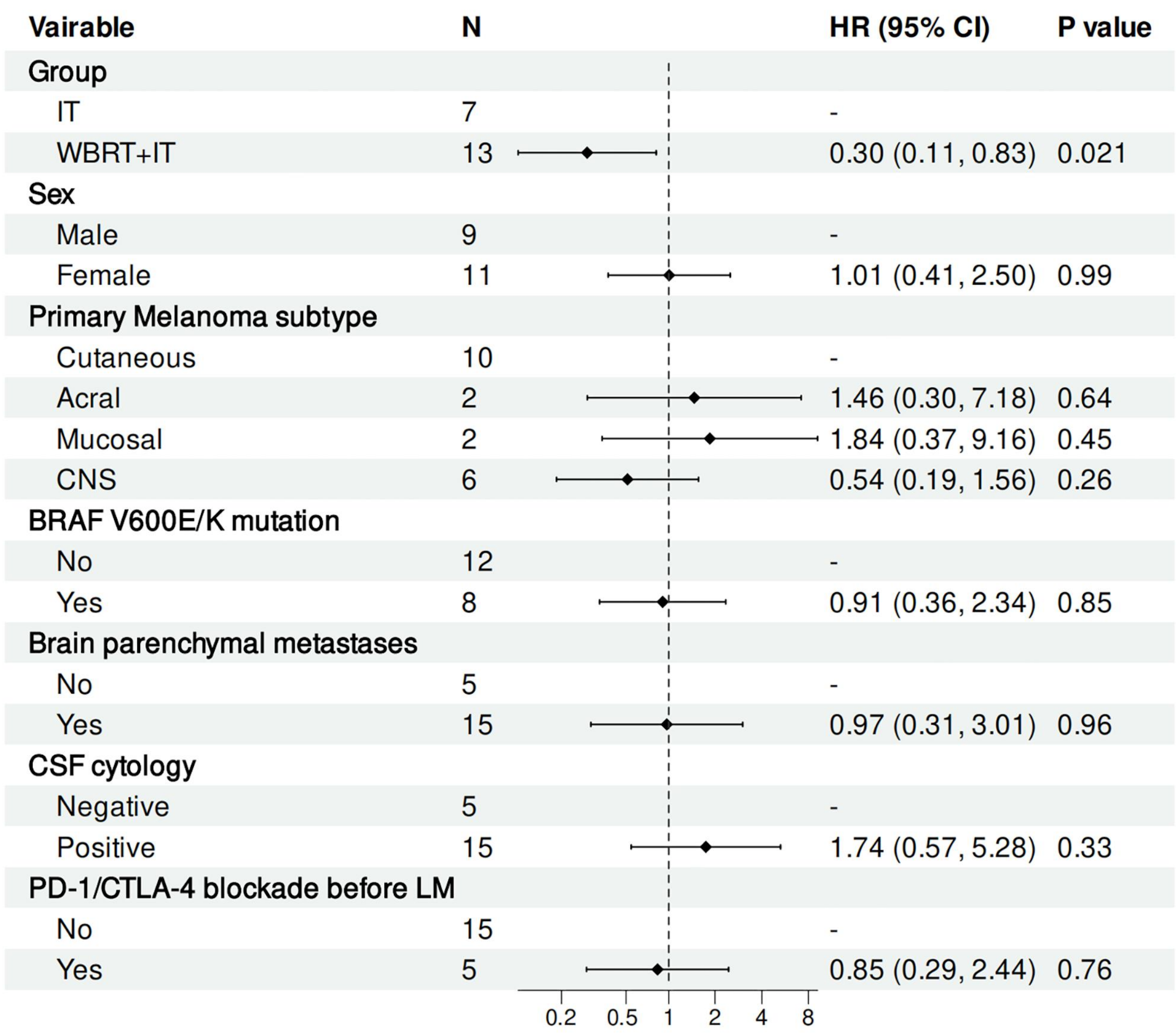

Supplementary Figure 1. Forest plot showing exploratory univariable associations between selected clinical or treatment-related variables and overall survival in patients with melanoma leptomeningeal metastasis treated with intrathecal anti-PD-1 therapy with or without WBRT. Hazard ratios and 95% confidence intervals are presented for descriptive purposes only. Abbreviations: HR, hazard ratio; CI, confidence interval; WBRT, whole-brain radiotherapy; IT, intrathecal therapy; CNS, central nervous system; CSF, cerebrospinal fluid; ICI, immune checkpoint inhibitor.

Supplementary Figure 2. Exploratory survival outcomes among patients with prior ICI exposure.

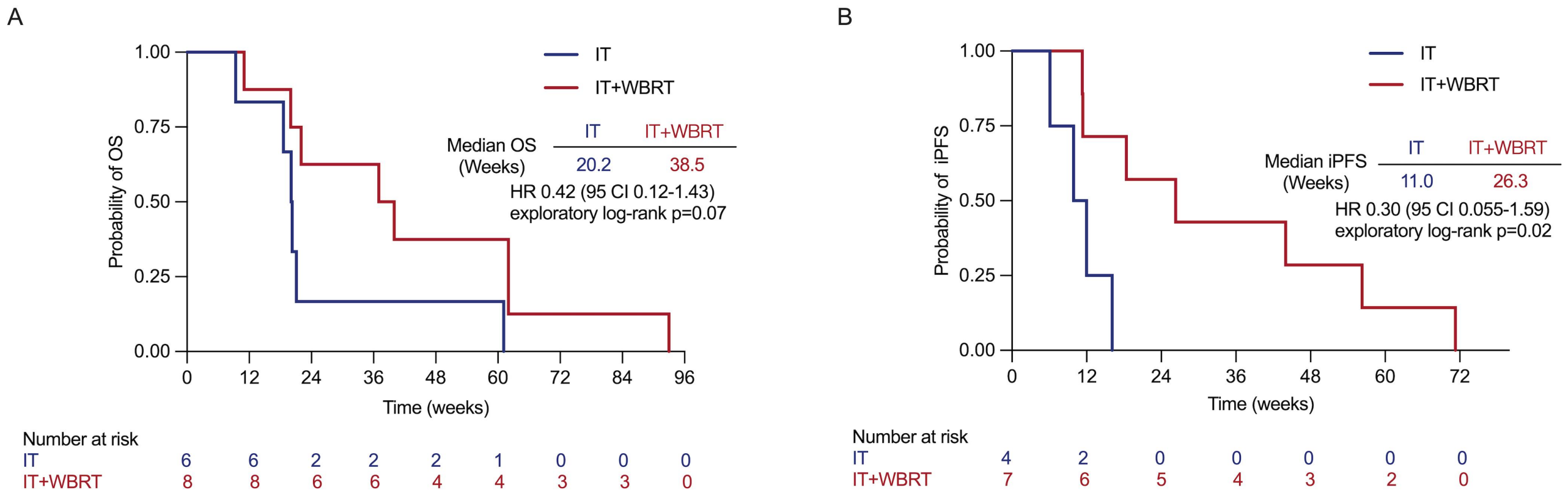

Supplementary Figure 2. Kaplan–Meier curves showing OS (A) and iPFS (B) according to treatment exposure among patients with prior ICI exposure. For iPFS, only patients with available post-treatment imaging follow-up were included: 4 patients in the intrathecal monotherapy group and 7 patients in the combination therapy group. Abbreviations: ICI, immune checkpoint inhibitor; IT, intrathecal therapy; WBRT, whole-brain radiotherapy; OS, overall survival; iPFS, intracranial progression-free survival.
